# Modulation of the Gut Microbiota in Rats by Hugan Qingzhi Tablets during the Treatment of High-Fat-Diet-Induced Nonalcoholic Fatty Liver Disease

**DOI:** 10.1155/2018/7261619

**Published:** 2018-12-23

**Authors:** Waijiao Tang, Xiaorui Yao, Fan Xia, Miaoting Yang, Zhijuan Chen, Benjie Zhou, Qiang Liu

**Affiliations:** ^1^Department of Pharmacy, Zhujiang Hospital, Southern Medical University, Guangdong, Guangzhou 510282, China; ^2^School of Traditional Chinese Medicine, Southern Medical University, Guangzhou 510515, China; ^3^Department of Pharmacy, Shantou Central Hospital, Affiliated Shantou Hospital of Sun Yat-sen University, Shantou, 515041 Guangdong, China; ^4^Department of Pharmacy, The Seventh Affiliated Hospital of Sun Yat-sen University, Shenzhen, 518107 Guangdong, China

## Abstract

**Background:**

Accumulative evidence showed that gut microbiota was important in regulating the development of nonalcoholic fatty liver disease (NAFLD). Hugan Qingzhi tablet (HQT), a lipid-lowering and anti-inflammatory medicinal formula, has been used to prevent and treat NAFLD. However, its mechanism of action is unknown. The aim of this study was to confirm whether HQT reversed the gut microbiota dysbiosis in NAFLD rats.

**Methods:**

We established an NAFLD model of rats fed with a high-fat diet (HFD), which was given different interventions, and measured the level of liver biochemical indices and inflammatory factors. Liver tissues were stained with hematoxylin-eosin and oil red O. Changes in the gut microbiota composition were analyzed using 16S rRNA sequencing.

**Results:**

The hepatic histology and biochemical data displayed that HQT exhibited protective effects on HFD-induced rats. Moreover, HQT also reduced the abundance of the *Firmicutes*/*Bacteroidetes* ratio in HFD-fed rats and modified the gut microbial species at the genus level, increasing the abundances of gut microbiota which were reported to have an effect on relieving NAFLD, such as *Ruminococcaceae*, *Bacteroidales_S24-7_group*, *Bifidobacteria*, *Alistipes*, and *Anaeroplasma*, and significantly inhibiting the relative abundance of *Enterobacteriacea*e, *Streptococcus*, *Holdemanella*, *Allobaculum*, and *Blautia*, which were reported to be potentially related to NAFLD. Spearman's correlation analysis found that *[Ruminococcus]_gauvreauii_group*, *Lachnoclostridium*, *Blautia*, *Allobaculum*, and *Holdemanella* exhibited significant (*p* < 0.001) positive correlations with triglyceride, cholesterol, low-density lipoprotein cholesterol, interleukin-6, interleukin-1*β*, tumor necrosis factor-*α*, and body weight and negative correlations with high-density lipoprotein cholesterol (*p* < 0.001). The *norank_f__Bacteroidales_S24-7_group* and *Alistipes* showed an opposite trend. Moreover, the HQT could promote flavonoid biosynthesis compared with the HFD group.

**Conclusion:**

In summary, the HQT has potential applications in the prevention and treatment of NAFLD, which may be closely related to its modulatory effect on the gut microbiota.

## 1. Introduction

Nonalcoholic fatty liver disease (NAFLD) is a common, multifactorial, and poorly understood liver disease with an increasing incidence globally [1]. Risk factors for NAFLD include a high-fat diet (HFD), a sedentary lifestyle, insulin resistance, and metabolic syndromes, such as obesity, dyslipidemia, and type 2 diabetes [2]. When treatment is delayed, NAFLD will progress to nonalcoholic steatohepatitis and even liver failure [3].

There are almost 10^14^ species of bacteria in the human intestinal tract. Gut microbiota of various types and high density has vital influences on gut health and is involved in the processes of food digestion, the defense for mucosal surfaces, and crosstalk with the host immune system.

The abundance and structure of the gut microbiota are significantly altered in patients with chronic liver diseases, such as nonalcoholic steatohepatitis [4, 5]. Additionally, the “gut-liver axis” theory has recently been proposed as an innovative concept contributing to NAFLD pathogenesis [6]. Patients with NAFLD usually have small intestinal bacterial overgrowth [7, 8], which can increase intestinal permeability and plasma levels of inflammatory markers, such as tumor necrosis factor- (TNF-) *α* and interleukin- (IL-) 8. Therefore, the gut microbiota may be a new potential therapeutic target for microbiota-related diseases.

The most common ways to regulate the gut microbiota include prebiotics, synbiotic supplements, and probiotics, or even traditional Chinese medicines (TCMs) [9, 10]. The gut microbiota is capable of performing a wide variety of metabolic transformations, such as giving the host the ability to digest phytochemical compounds [11]. The majority of TCMs contain phytochemical ingredients, such as flavonoids, alkaloids, polysaccharides, and saponins, which are not only easily metabolized by gut microbiota but also often administered orally [12, 13], suggesting that these medicines can directly act on gut microbiota and restore its homeostasis. For example, berberine, a clinically effective drug treatment for NAFLD that contains isoquinoline alkaloid, has recently been shown to exert its actions by modulating the gut microbiota [14]. Tea polyphenols and saponins could increase the diversity of gut microbiota and altered its structure [15, 16].

The Hugan Qingzhi tablet (HQT) has a long history of use in alleviating NAFLD in clinical practice. In our previous in vitro and in vivo studies, it was confirmed that the HQT exerted lipid-lowering and anti-inflammatory effects on NAFLD [17, 18]. However, the relevant mechanism involved in its effect on ameliorating NAFLD requires further investigation. In our isobaric tags for relative and absolute quantitation-based proteomics experiments on livers from NAFLD rats, a Kyoto Encyclopedia of Genes and Genomes (KEGG) pathway enrichment analysis showed that the gut microbiota is closely related to the pathogenesis of NAFLD [19]. The major Chinese medicines in the HQT are Rhizoma Alismatis, Fructus Crataegi, Pollen Typhae, Folium Nelumbinis, and Radix Notoginseng (Additional files 1: Table 1). Among them, Fructus Crataegi and Pollen Typhae contain large amounts of flavonoids, such as quercetin and rutin [20, 21]. Flavonoids are metabolized by the enzymes produced by the gut microbiota, thus affecting the bioavailability of flavonoids in the human body [22], suggesting that the HQT may interact with the gut microbiota and contribute to NAFLD. However, direct evidence for the effects of the HQT on modulating the gut microbiota is still lacking. Therefore, the 16S rRNA gene sequencing technique is used in this research to compare the structural changes of gut microbiota caused by HQT in HFD-driven NAFLD rats. This research would offer a solid basis for the regulation of gut microbiota structure by HQT therapies, which will help researchers to further comprehend the interaction between the host and microorganisms during the therapeutical process of NAFLD and the mechanism of action of HQT in this process.

## 2. Materials and Methods

### 2.1. Plant Material and Preparation of the HQT

HQTs were provided by Zhujiang Hospital, Southern Medical University (SMU) (Guangzhou, China). 70% ethanol (1 : 6, *m*/*v*) was utilized to impregnate 30% Rhizoma Alismatis, 30% Fructus Crataegi, 20% Folium Nelumbinis, and 15% Pollen Typhae for about 1.5 hours, and then the measure of reflux was used to extract those materials for 2 hours; this process was repeated three times. It was estimated that the productivity of dried extracts is about 14.45% (*m*/*m*). After that, 5% of Panax notoginseng was squashed and screened and then mixed with the dried extracts to generate HQT. In previous studies, we have stated the approach for carefully identifying and quantifying the main components of HQT [18, 23].

### 2.2. Analysis of HQT by UHPLC-QqQ-MS

HQT (0.50 g) was precisely weighed and put into extraction by an ultrasonic water bath (30°C) with 50 mL of methanol-water solution (1 : 1, *v*/*v*) for 30 minutes. Next, filtration of the extract was performed using a 0.22 *μ*m syringe filter. The extracted filtrate was utilized as an experimental solution and analyzed by ultra-high-performance liquid chromatography-triple-quadrupole mass spectrometry (UHPLC-QqQ-MS).

Chromatographic analyses were carried out under the Agilent 1290-6460 series UHPLC system (Agilent Technologies, Santa Clara, USA). The chromatographic fractionation was carried out using 0.1% formic acid and water as mobile phase A and acetonitrile as mobile phase B in a gradient manner at a temperature of 30°C using a Waters ACQUITY UPLC C18 column (2.1 × 100 mm, 1.7 *μ*m) (Waters, Milford, MA, USA). The gradient elution program was as follows: 0–6 min, 18–20% B; 6–7 min, 20–25% B; 7–7.01 min, 25–50% B; 7.01–10 min, 50–90% B; and 10–12 min, 90–90% B. The sample injection size was 2 *μ*L, and the flow rate was 0.35 mL/min. The mass spectrometry analysis was performed on an Agilent 6460 QqQ-MS (Agilent Technologies, Santa Clara, USA) equipped with an electrospray ionization (ESI) source. Then, the ESI source was operated in positive and negative ionization modes. The MS and MS/MS spectra of the 12 compounds were attained by immediately infusing every normalized solution.

### 2.3. Animals and Treatment

Animal testing mentioned in this research was conducted at the animal facilities of the Animal Ethics Committee of SMU which is consistent with references in the Guide for the Care and Use of Laboratory Animals of China. Male Sprague–Dawley (SD) rats (180–220 g) were supplied by the SMU Animal Experiment Center (Guangzhou, China, quality certificate number: SCXK (Yue) 2011-0015). All endeavors were made to minimize the sufferings of the animals involved in the experiment. After one-week acclimation, 24 male SD rats (8 weeks old, specific pathogen-free) were stochastically divided into 3 groups of 8 each. One group of rats was conventionally raised with a normal-fat diet (NFD), one group was fed a HFD, and another group was fed a HFD plus the HQT in a 1.08 g/kg BW HQT suspension [18]. The food intake of each group is at the same level. The group of rats that were fed a HFD was offered with chow containing 1.2% cholesterol, 15% lard, 20% sucrose, 0.2% sodium cholate, 0.6% dicalcium phosphate, 0.4% limestone, 10% casein, and 0.4% premix mixed with the NFD. Each group was given the appropriate therapy daily, and body weight (BW) was recorded once a week. Samples were collected weekly from the feces of 15 rats from the NFD, HFD, and HQT groups before they were sacrificed. Unluckily, the feces gathered from the other three animals were inadequate, so the gut microbiota of these three animals was no longer analyzed.

### 2.4. Liver Histology and Serum Analysis

At the end of the 12-week trial, all three groups of rats were executed and their livers were taken out and kept at −80°C for subsequent histological and lipid analysis, including hematoxylin-eosin (HE) and oil red O staining and triglyceride (TG), cholesterol (CHOL), low-density lipoprotein cholesterol (LDL-C), and high-density lipoprotein cholesterol (HDL-C) tests. Morphological and pathological analyses of HE staining in rat liver were conducted, and statistical evaluation was made regarding the NAFLD activity score (NAS) [24]. The Olympus Image-Pro Plus 6.0 software was used to conduct the quantitative analysis and calculate the oil red O staining areas. Additionally, concentrations of proinflammatory cytokines, including interleukin-6 (IL-6), tumor necrosis factor-*α* (TNF-*α*), and IL-1*β*, in hepatic homogenates were quantified utilizing enzyme-linked immunosorbent assay kits, under the instructions of the producer (Mutisciences, Hangzhou, China).

### 2.5. DNA Extraction, PCR Amplification, and Illumina MiSeq Sequencing

E.Z.N.A.® Stool DNA Kit (Omega Bio-Tek, Norcross, GA, USA) was utilized to extract microbial DNA from fecal samples. The V3-V4 region of the bacterial 16S rRNA was intensified by PCR (95°C for 2 min, followed by 25 cycles at 95°C for 30 s, 55°C for 30 s, and 72°C for 30 s and a final extension at 72°C for 5 min) using primers [25] 5′-ACTCCTACGGGAGGCAGCAG-3′ for 338F and 5′-GGACTACHVGGGTWTCTAAT-3′ for 806R. The PCR reaction was implemented in a 20 *μ*L blend comprising 4 *μ*L of 5× FastPfu buffer, 2 *μ*L of 2.5 mM dNTPs, 0.8 *μ*L of each primer (5 *μ*M), 0.4 *μ*L of FastPfu polymerase, and 10 ng of template DNA. The amplicon was extracted from 2% agarose gel and purified by the AxyPrep DNA Gel Extraction Kit (Axygen Biosciences, Union City, CA, USA), and QuantiFluor™-ST (QuantiFluor) was used for quantification. The purified amplifier was merged at the same molar concentration, and paired-end sequencing (2 × 300) was performed on the Illumina MiSeq platform using the standard protocol. PE amplicon libraries were established, and sequencing was conducted utilizing the Illumina MiSeq platform at Majorbio Bio-Pharm Technology Co. Ltd., Shanghai, China. Raw fastq files were demultiplexed and quality-filtered utilizing FLASH and Trimmomatic.

### 2.6. Bioinformatics and Statistical Analysis

The operational taxonomic units (OTUs) that reached a 97% nucleotide similarity level were subjected to alpha-diversity analyses using mothur software [26]. Beta-diversity measurements were calculated as previously described [27], and principal coordinate analyses (PCoA) on the basis of OTU abundance and distance were verified. R package was utilized for the visualization of bacterial community classification and distribution. For linear discriminant analysis effect size (LEfSe) [28], biological relevance and statistical significance were taken into account, and identification was performed to differentially represent the level of classification among the three groups. Microbial functions were forecasted utilizing phylogenetic investigation of communities by reconstruction of unobserved state (PICRUSt) [29]. The predicted genes and their functions are in line with the KEGG database and compared to the STAMP software (http://kiwi.cs.dal.ca/Software/STAMP) [30].

### 2.7. Statistical Analysis

All the results are presented as means ± SD. Benjamini-Hochberg FDR (*p* < 0.05) correction and two-sided Welch's test were utilized in the examination of differences in microbial functions between two groups. The one-way analysis of variance (ANOVA) was used to analyze the mean values in the three groups. GraphPad Prism software (version 6.02) was used to perform the statistical analysis. After the comparison with the control group, it is obvious that all the consequences are considered to be statistically significant at *p* < 0.05.

## 3. Results

### 3.1. The UHPLC-QqQ-MS Analysis of the HQT

**Figure 1:**
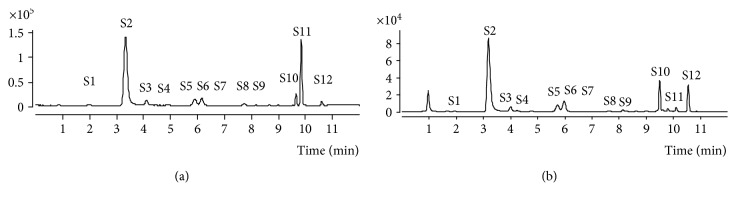
The UHPLC-QqQ-MS analysis of the HQT. (a) The UHPLC-QqQ-MS analysis of standard reference materials. Epicatechin (S1), nuciferine (S2), typhaneoside (S3), rutin (S4), heterosine lisu-3-o-new hesperidin (S5), hyperoside (S6), isoquercetin (S7), notoginsenoside R1 (S8), quercetin (S9), isorhamnetin (S10), alisol A 24-acetate (S11), and 23-O-acetylalisol B (S12). (b) The UHPLC-QqQ-MS analysis of the HQT.

UHPLC-QqQ-MS spectrometry was used to characterize the chemical HQT composition. The total ion current chromatograms of the HQT are shown in Figure 1. Twelve major compounds were identified and quantified by a comparison with reference standards. Their chemical names are epicatechin (S1), nuciferine (S2), typhaneoside (S3), rutin (S4), heterosine lisu-3-o-new hesperidin (S5), hyperoside (S6), isoquercetin (S7), notoginsenoside R1 (S8), quercetin (S9), isorhamnetin (S10), alisol A 24-acetate (S11), and 23-O-acetylalisol B (S12).

### 3.2. The HQT Attenuates HFD-Induced NAFLD

**Figure 2:**
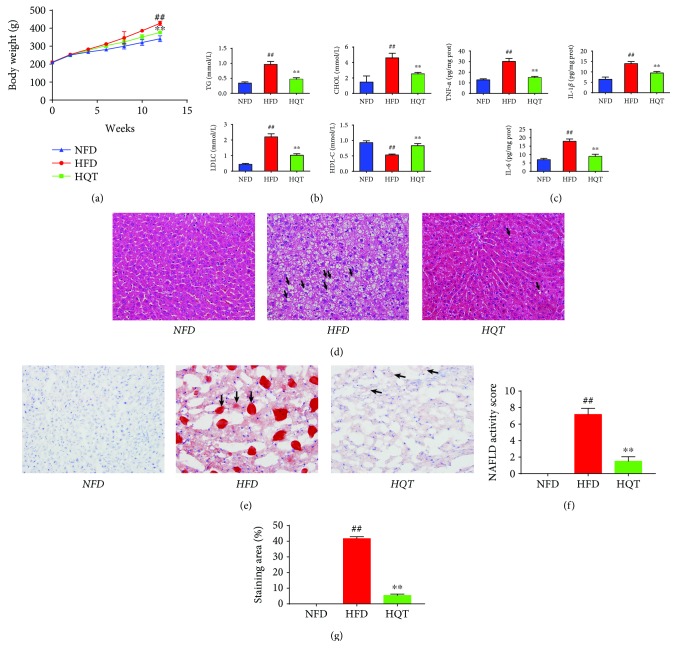
Effects of the HQT on the BW (a); hepatic TG, CHOL, HDL-C, and LDL-C levels (b); hepatic IL-6, IL-1*β*, and TNF-*α* levels (c); the HE-stained liver tissue (d); oil red O-stained liver tissue (e) from rats in the NFD, HFD, and HQT groups the results of the NAFLD activity score of liver HE staining (f); and the quantitative results of the oil red O staining (g). *n* = 8 rats per group. Values are presented as the means ± SD for each group. ^∗^ *p* < 0.01, ^∗∗^ *p* < 0.01 compared with the HFD group. ^#^ *p* < 0.01, ^##^ *p* < 0.01 compared with the NFD group.

After a 12-week intervention period, rats in the HFD group weighed significantly more than rats in the NFD group (Figure 2(a)). As shown in Figure 2(b), the liver TG and CHOL levels in the HQT group were much lower than those in the HFD group. The liver HDL-C level exhibited the opposite trend to the TG and CHOL levels, which were significantly increased. HFD-fed rats displayed a higher liver LDL-C level than the NFD group, which was significantly reduced after the treatment with the HQT. Compared with the NFD group (Figure 2(c)), IL-6, IL-1*β*, and TNF-*α* levels were drastically elevated in the HFD group (*p* < 0.01), and the levels observed in the HQT group were significantly decreased compared with those in the HFD group (*p* < 0.01).

As shown in Figure 2(d), extensive micro/macrovesicular steatosis was detected in the hepatocytes of HFD rats, and we also observed macrovesicular steatosis, steatohepatitis changes, inflammation, and massive infiltration of inflammatory cells around the central vein of hepatocytes in the HFD rats. In contrast, the HQT ameliorated these morphological changes. The analysis of the NAFLD activity score showed that in HFD rats the score was significantly increased (Figure 2(f)). However, HQT treatment could significantly inhibit such increase. Moreover, hepatocyte lipid accumulation was significantly decreased in the HQT group compared with the HFD group (Figure 2(e)). Quantification of oil red O staining showed that lipid deposition occurred in nearly 41.87% in HFD rats' liver tissue, and HQT treatment significantly reduced HFD-induced liver lipid deposition (Figure 2(g)). It is clear that HQT significantly prevented rats' liver lipid deposition caused by HFD feeding for up to 12 weeks. Based on these results, the HQT exerted a protective effect on HFD-fed rats by lowering the levels and inhibiting inflammation.

### 3.3. Response of the Gut Microbiota Structure to the HQT in HFD-Fed Rats

16S rRNA gene sequencing was used to investigate whether HQT had an effect on the structure of gut microbiota in HFD-fed rats. Good's coverage beyond 99.5% demonstrated an adequate sequencing depth for all samples (Additional files 2: Table 2). With quality control, 1,663,537 reads (average of 36,967 sequences per sample) were delineated into 794 OTUs. Rarefaction and Shannon index analyses showed that most of the diversity and rare new phylotypes could be covered by the sequencing depth (Additional files 3: Figure 1).

**Figure 3:**
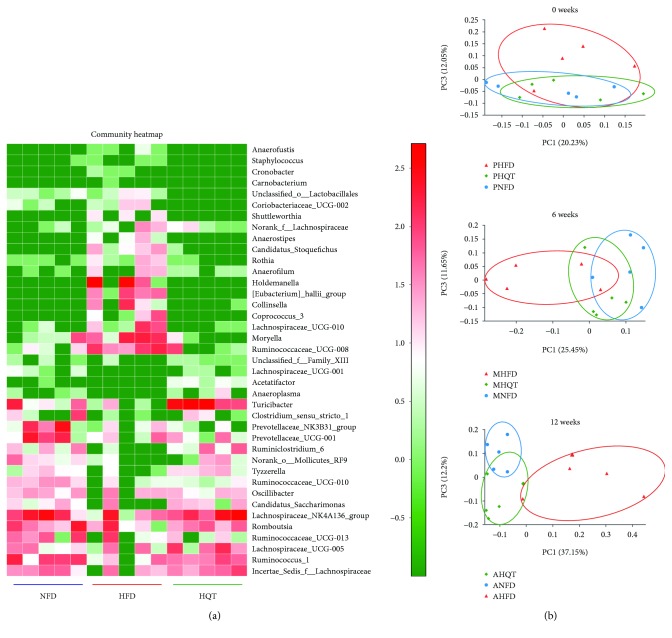
The community heatmap of the NFD, HFD, and HQT groups at week 12 (a). The PCoA analyses performed at weeks 0, 6, and 12 in the NFD, HFD, and HQT groups (b). P = prophase; M = metaphase; A = anaphase. *n* = 5 rats per group.

As shown in the community heatmap diagram, the abundance of 39 genera was significantly different among the three groups (Figure 3(a)). The HFD group exhibited higher abundances of *Moryella*, *[Eubacterium]_hallii_group*, *Collinsella*, and *Ruminococcaceae_UCG-008* than the NFD and HQT groups. In addition, the HFD group displayed lower abundances of the genera *Ruminiclostridium_6*, *Tyzzerella*, *norank_o_Mollicutes_RF9*, and *Candidatus Saccharimonas* than the NFD and HQT groups. On the basis of the unweighted UniFrac distance calculation, the PCoA of *β*-diversity in the gut microbiota (Figure 3(b)) did not reveal a significant difference in the gut microflora among the three groups before the experiment started (at week 0). After administering the HFD and HQT interventions, the gut microbiota of the HFD and the HQT groups gradually separated from that of the NFD rats in the middle of the experiment (at week 6). At the end of the experiment (at week 12), the gut microbiota of rats in the HFD group was completely separated from that of the NFD group while the gut microbiota of the HFD and HQT groups was separated, and the distance of the gut microbiota between the HQT group and the NFD group was shorter. Therefore, we speculated that after the HFD and HQT interventions, the gut structure in the three groups of rats became significantly different.

**Figure 4:**
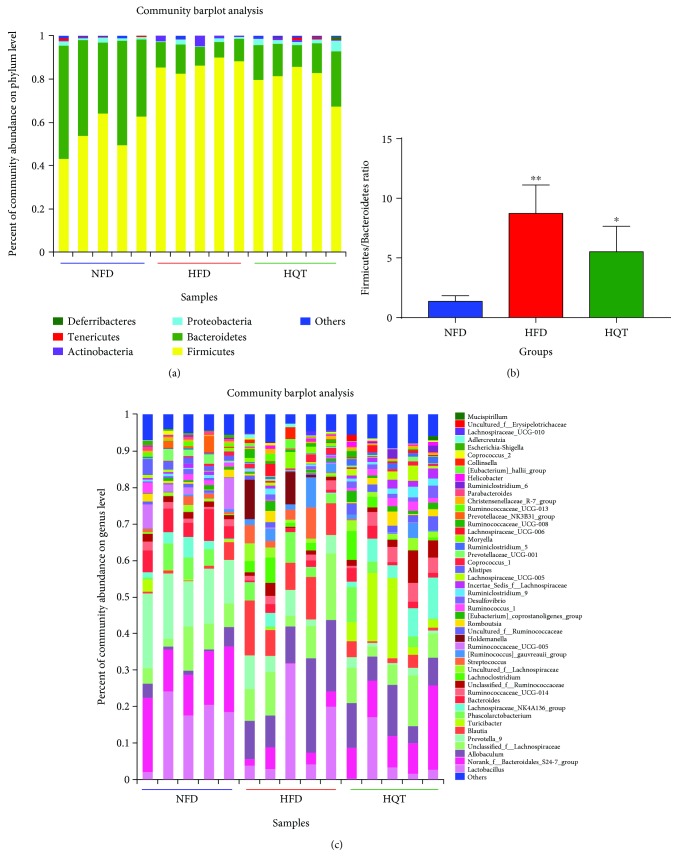
Bacterial composition of the different communities at the phylum level in the NFD, HFD, and HQT groups at week 12 (a). The Firmicutes-to Bacteroidetes ratio in the NFD, HFD, and HQT groups at week 12 (b). Bacterial composition of the different communities at the genus level in the NFD, HFD, and HQT groups at week 12 (c). *n* = 5 rats per group. Values are presented as the means ± SD for each group. ^∗^ *p* < 0.01, ^∗∗^ *p* < 0.01 compared with the HFD group. ^#^ *p* < 0.01, ^##^ *p* < 0.01 compared with the NFD group.

The composition of the gut microbiota at both the phylum and genus levels was analyzed to determine which types of bacteria were affected by HFD and HQT intake. At the phylum level, *Bacteroides*, *Firmicutes*, and *Proteobacteria* were the main components of the gut microbiota in the rat feces (Figure 4(a)). Compared with the NFD group, an increase in *Firmicutes* and a decrease in *Bacteroidetes* were observed in the HFD rats. Compared with the HFD group, the abundance of *Firmicutes* was decreased in the HQT group but the abundance of *Bacteroidetes* was increased (Figure 4(b)). However, based on the importance of the *Firmicutes*-to-*Bacteroidetes* (F/B) ratio, a significantly higher ratio was observed in the HFD group (*p* < 0.01) than in the NFD group and a lower ratio was observed in the HQT group (*p* < 0.05) than in the HFD group. As shown in the results of the genus-level analyses (Figure 4(c)), compared with the NFD group, reduced abundance of *Prevotella_9*, *norank_f_Bacteroidales_S24-7_group*, *Bacteroides*, and *Ruminococcus_1* and increased abundance of *Allobaculum*, *Blautia*, *[Ruminococcus]_gauvreauii_group*, and *Holdemanella* were observed in the HFD group. Compared with the HFD group, *norank_f_Bacteroidales_S24-7_group*, *Turicibacter*, *Lachnospiraceae_NK4A136_group*, and *Ruminococcus_1* were increased, but *Blautia*, *Prevotella_9*, and *Holdemanella* were reduced in the HQT group.

### 3.4. Key Phylotypes of Gut Microbiota Modulated by the HQT

**Figure 5:**
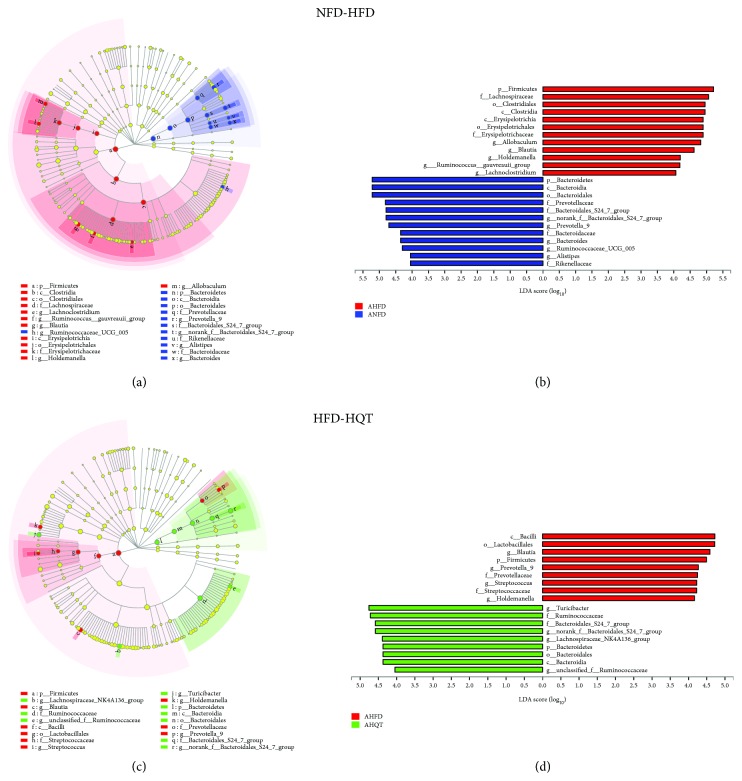
Key bacterial alterations in response to the HFD and HQT treatments. (a) Cladogram generated by the LEfSe analysis (LDA = 4) showing enriched taxa in 12-week feces from the NFD (blue) and HFD (red) groups. (b) LDA scores of enriched taxa shown in (a). (c) Cladogram generated by the LEfSe analysis (LDA = 4) showing enriched taxa in 12-week feces from the HFD (red) and HQT (green) groups. (d) LDA scores of enriched taxa shown in (c). *n* = 5 rats per group. Notes: A = anaphase (ANFD, AHFD, and AHQT).

Based on the linear discriminant analysis (LDA) values of 4 (Figure 5) and 2 (Additional files 4: Figure 2), the NFD, HFD, and NFD groups were statistically analyzed by using the LEfSe method to further examine the differences in the abundance of bacterial species in each group.

As shown in the taxonomic cladograms, the LEfSe analysis demonstrated the modulatory effects of the HQT on different taxonomic levels of the gut microbiota in rats fed with the HFD (Figure 5 and Additional files 4: Figure 2). Compared with the NFD group, the abundances of *Bacteroides*, *norank_f__Bacteroidales_S24_7_group*, *Butyricimonas*, *Parabacteroides*, *Alloprevotella*, *Prevotella*_9, and *unclassified_o__Bacteroidales* belonging to the *Bacteroidetes* phylum were decreased in the HFD group. Additionally, the HFD restrained the growth of *Anaerotruncus*, *Ruminiclostridium*, *Ruminiclostridium*_5, *Ruminiclostridium*_6, *Ruminococcaceae_UCG_007*, *Ruminococcaceae_UCG_009*, *Ruminococcus*_1, *unclassified_f__Ruminococcaceae*, and *uncultured_f__Ruminococcaceae*, which belong to the family *Ruminococcaceae*. In addition, the abundance of *Cronobacter*, which belongs to the family *Enterobacteriaceae*, was obviously increased in the HFD group compared with the NFD group.

Compared with the HFD group, the abundance of *norank_f__Bacteroidales_S24_7_group*, *Alistipes*, and *Cronobacter* was increased in the HQT group. Additionally, compared with the HFD group, the HQT increased the growth of *Anaerotruncus*, *Ruminiclostridium*, *Ruminiclostridium_5*, *Ruminiclostridium_6*, *Ruminococcaceae_UCG_007*, *Ruminococcaceae_UCG_009*, *Ruminococcus_1*, *unclassified_f__Ruminococcaceae*, and *uncultured_f__Ruminococcaceae*, which belong to the family *Ruminococcaceae*. Additionally, the abundances of the genera *Staphylococcus*, *Streptococcus*, and *unclassified_o__Lactobacillales*, which belong to *Bacilli*, were decreased, while the abundances of *Anaeroplasma*, *Bilophila*, *Desulfovibrio*, and *Bifidobacterium* were increased in the HQT group compared with the HFD group.

Based on these results, the HQT could modulate the gut microbiota of HFD-fed rats, resulting in a microbiota composition similar to that of NFD rats.

### 3.5. Associations between the Gut Microbiota Composition and NAFLD Phenotypes

**Figure 6:**
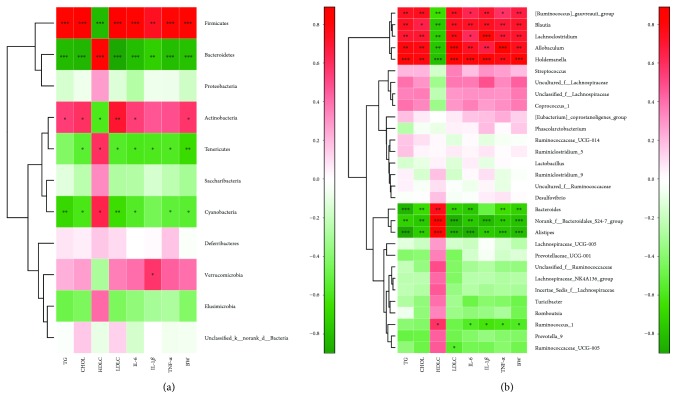
The correlations between the relative abundance of the gut microbial community at the phylum level and vital metabolic parameters linked to NAFLD in the NFD, HFD, and HQT groups at week 12 (a). The correlations between the relative abundance of the gut microbial community at the genus level and vital metabolic parameters linked to NAFLD in the NFD, HFD, and HQT groups at week 12 (b). *n* = 5 rats per group. ^∗^0.01 < *p* ≤ 0.05, ^∗∗^0.001 < *p* ≤ 0.01, ^∗∗∗^ *p* ≤ 0.001.

The correlations between the relative abundance of the gut microbial community and important metabolic parameters associated with NAFLD were presented in Spearman's correlation heatmap. Pearson's correlation analysis was used to determine the correlation of each microbial level. At the phylum level (Figure 6(a)), significant correlations were observed between the parameters tested and the relative abundances of *Firmicutes*, *Bacteroidetes*, *Actinobacteria*, *Tenericutes*, and *Cyanobacteria*. However, both *Bacteroidetes* and *Cyanobacteria* exhibited a definite negative correlation with TG, CHOL, LDL-C, IL-6, IL-1*β*, TNF-*α*, and BW and a positive correlation with HDL-C while *Firmicutes* showed a positive correlation with TG, CHOL, LDL-C, IL-6, IL-1*β*, TNF-*α*, and BW and a negative correlation with HDL-C.

At the genus level (Figure 6(b)), *[Ruminococcus]_gauvreauii_group*, *Lachnoclostridium*, *Blautia*, *Allobaculum*, and *Holdemanella* exhibited significant (*p* < 0.001) positive correlations with TG, CHOL, LDL-C, IL-6, IL-1*β*, TNF-*α*, and BW and negative correlations with HDL-C. The *norank_f__Bacteroidales_S24-7_group* and *Alistipes* showed significant (*p* < 0.001) negative correlations with TG, CHOL, LDL-C, IL-6, IL-1*β*, TNF-*α*, and BW and positive correlations with HDL-C. *Bacteroides* displayed the same trend, except for the IL-1*β* index.

### 3.6. Predictions of Gut Microbiota Functions in the NFD, HFD, and HQT Groups

The mechanism by which gut microbes exert their biological effects is closely related to the function of the genes encoded in the gut microbiome. Therefore, we predicted corresponding changes in gene abundance and metabolic pathways using PICRUSt and calculated the changes in functional pathways between groups using STAMP software.

**Figure 7:**
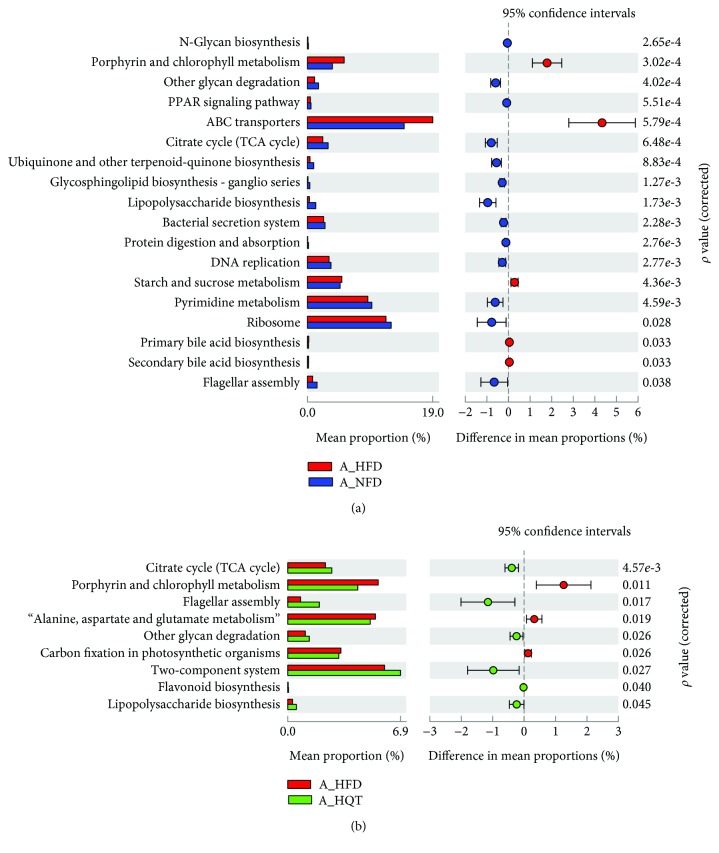
Comparisons of functional pathways in microbes from the NFD and HFD groups at 12 weeks (a) and in the HFD and HQT groups at 12 weeks (b). *n* = 5 rats per group. The pathways shown in the figure were predicted by PICRUSt. Noted: A = anaphase.

In the comparison of the HFD and NFD groups, the microbiota in the former comprised more functions involved in metabolic pathways involving ATP-binding cassette (ABC) transporters, primary bile acid synthesis, and secondary bile acid synthesis than the microbiota in the latter. In contrast, the NFD group included functions involved in the tricarboxylic acid (TCA) cycle, flagellar assembly, other glycan degradation, two-component system, and lipopolysaccharide biosynthesis (Figure 7(a)). Regarding the comparison of the HFD and HQT groups, the microbiota in the HFD group displayed more functions in porphyrin and chlorophyll metabolism, alanine aspartate and glutamate metabolism, and carbon fixation in photosynthetic organisms than the microbiota in the HQT group. However, similar to the NFD group, the HQT group had a greater number of functions in the TCA cycle, flagellar assembly, other glycan degradation, two-component system, lipopolysaccharide biosynthesis, and flavonoid biosynthesis (Figure 7(b)).

## 4. Discussion

In this study, rats fed with a HFD gained significantly more weight than rats fed with a NFD. Additionally, pathological indicators of NAFLD and liver biochemical markers and inflammatory factor indexes confirmed the validity of the model. The HQT sufficiently reduced the accumulation of lipids, such as TG, CHOL, HDL-C, and LDL-C, and inflammation indicators, such as TNF-*α*, IL-6, and IL-1*β* levels, indicating its lipid-lowering and anti-inflammatory effects on alleviating NAFLD progression.

According to the UHPLC-QqQ-MS analysis, the HQT contained flavonoids, such as typhaneoside, rutin, quercetin, heterosine lisu-3-o-new hesperidin, hyperoside, and isoquercetin, which were mainly obtained from Pollen Typhae and Fructus Crataegi [20, 21]. Orally administered flavonoids, such as rutin, are not detectable in blood, but the aglycone form quercetin has been observed in blood [31]. In fact, the quercetin glucoside or quercetin has been reported to be directly absorbed by the small intestine [31]. Likewise, other flavonoids, such as hesperidin, also share similar metabolic fates in the human body [32]. The detection of these components also implied that the HQT may treat NAFLD by regulating the gut microbiota.

Concomitant with the improved clinical index of NAFLD, we observed an altered microbial composition induced by the HQT and HFD. Based on the PCoA, the consumption of HFD for up to 12 weeks shifted the gut microbiota structure in NAFLD rats. The HQT showed to reverse the HFD-induced structural variations.

Certain studies [33, 34] have observed a close correlation between obesity and an increase in the intestinal *Firmicutes*-to-*Bacteroidetes* (F/B) ratio in both mouse experiments and clinical trials. In this study, the F/B ratio was significantly elevated in the HFD group compared with the NFD group, while the F/B ratio was significantly decreased in the HQT group compared with the HFD group.

Compared with the HFD group, *norank_f__Bacteroidales_S24_7_group*, *Anaeroplasma*, *Bifidobacterium*, *Bilophila*, *Desulfovibrio*, and most of the genera belonging to family *Ruminococcaceae*, such as *Anaerotruncus*, *Ruminiclostridium*, *Ruminiclostridium_5*, *Ruminiclostridium_6*, *Ruminococcaceae_UCG_007*, *Ruminococcaceae_UCG_009*, *Ruminococcus_1*, *unclassified_f__Ruminococcaceae*, and *uncultured_f__Ruminococcaceae*, were markedly enriched after HQT treatment. The *norank_f__Bacteroidales_S24_7_group* and *Ruminococcaceae* are butyrate-producing bacteria. Butyrate is a short-chain fatty acid produced from resistant starch, dietary fiber, and low-digestible polysaccharides by the microbiota in the colon and distal small intestine via fermentation [35–37]. As shown in the study by Endo et al. [38], butyrate-producing probiotics reduce NAFLD progression in rats. Zhou et al. [35] also verified that sodium butyrate attenuates HFD-induced steatohepatitis in mice by improving the gut microbiota and gastrointestinal barrier. These findings suggest an important role for the butyrate-producing bacteria in the efficacies of the HQT.

In 1998, the concept of an “intestine-liver axis” was proposed, suggesting that intestinal barrier function is damaged after the intestinal tract is injured. Large quantities of bacteria and endotoxins from the intestine subsequently enter the liver through the portal venous system to activate Kupffer cells and liver cells that release a series of inflammatory cytokines, such as TNF-*α*, IL-1*β*, and IL-6, further damaging the liver [6]. In our experiments, the abundance of some intestinal endotoxin- (LPS) rich bacteria was significantly increased in the HFD group, and the HQT reversed this trend. LPS is the main ingredient of the outer membrane of Gram-negative bacteria, and it is the endotoxin that goes into the circulation causing inflammation [39]. According to the research of Cani et al. [40], mice fed with a HFD for as short term as 2 to 4 weeks exhibited a significant increase in plasma LPS. LPS derived from the members of the families *Enterobacteriaceae* and *Desulfovibrionaceae*, in the phylum *Proteobacteria*, exhibits a 1000-fold increase in endotoxin activity compared to LPS derived from the family *Bacteroidaceae*, in the phylum *Bacteroidetes* [41]. In our experiments, the abundance of *Cronobacter*, which belongs to the family *Enterobacteriaceae*, was significantly increased in the HFD group compared with the HQT group. The abundances of *Desulfovibrio* and *Bilophila* which belong to the family *Desulfovibrionaceae* were increased in the HQT group, which was unexpected, and this result indicated that the HQT might selectively alter the abundance of some bacteria associated with inflammation.

In our studies, the HQT group exhibited an increase in the abundance of *Bifidobacterium*, and the abundance of this genus was significantly decreased in the HFD group. This finding is consistent with the results of a previous study [40], showing that a HFD decreases the abundance of *Bifidobacteria* in mice and increases the levels of LPS, TNF-*α*, IL-1, and IL-6, leading to intestinal mucosa infiltration. It is also reported by Nobili et al. [42] that Bifidobacteria seem to have a protective effect on the development of NAFLD and obesity in the gut microbiome of NAFLD children. In those studies, *Bifidobacteria* reduced LPS levels in mice and improved mucosal barrier function [43, 44]. Our results showed that the HQT group excreted significantly higher proportions of Bifidobacterium than the HFD group (*p* < 0.05) and this may relate to its protective role in NAFLD.

In addition, the genera *Alistipes* and *Anaeroplasma* in the HQT group were also more abundant than those in the HFD group. It is worth noting that *Alistipes* belongs to the family *Rikenellaceae*, which was also decreased in patients with NAFLD in a recent study [45]. Clarke [46] reported a significant decrease in the abundance of *Anaeroplasma* in obese mice compared with lean mice.

The abundances of *Streptococcus* which belong to *Bacilli* were significantly increased in the HFD group compared with the HQT group. Compared with healthy subjects, NAFLD patients show an increase in the percentage of bacteria from the pathogenic Streptococcus, which may induce persistent inflammation of the intestinal mucosa and is associated with inflammatory bowel disease [47, 48]. In our experiments, it is also found that some genera such as *Holdemanella* and *Allobaculum* were higher in the HFD group than in the HQT group. Furthermore, the genera *Holdemanella* and *Allobaculum* exhibited significant (*p* < 0.001) positive correlations with TG, CHOL, LDL-C, IL-6, IL-1*β*, TNF-*α*, and BW and negative correlations with HDL-C. Brahe et al. [49] reported that *Holdemanella* is associated with an unhealthy fasting serum lipid level. In our experiments, the abundance of *Holdemanella* was extremely low in the NFD and HQT groups but was substantially increased in the HFD group, thus suggesting that the high abundance of *Holdemanella* may be an important cause of NAFLD. Low-dose penicillin causes weight gain in infant mice because it can alter the proportions of dominant bacteria, such as reducing the abundance of *Allobaculum*, in newborn or infant mice. Thus, researchers postulate that the increased abundance of *Allobaculum* can help infant mice resist the development of obesity [50]. However, in our experiment, the abundance of *Allobaculum* was substantially increased in the HFD group compared to the HQT group, which may be related to the use of adult rats in the present study. In addition, the genus *Blautia* exhibited significant (*p* < 0.001) positive correlations with TG, CHOL, LDL-C, IL-6, IL-1*β*, TNF-*α*, and BW and a negative correlation with HDL-C. Several studies have observed a correlation between *Blautia* and obesity. For example, Goffredo et al. reported a positive correlation between the abundance of *Blautia* and obesity in American youth and verified that the level of acetate, which is the product of *Blautia*, is associated with body fat partitioning and hepatic lipogenesis [51].

The expression of genes encoding ABC transporters is reportedly increased in the fecal microbiome of mice fed with a HFD compared to mice fed with standard chow or a low-fat diet [52, 53]. In our experiment, the levels of the ABC transporter genes were significantly increased in the HFD group compared with the NFD group. Interestingly, the HFD group also displayed a significant increase in the levels of the genes involved in primary bile acid synthesis and secondary bile acid synthesis compared with NFD rats. HFD-induced bile acid secretion was originally thought to be a driving force affecting the composition of obesity-related gut microbiota [54], and rats fed with cholic acid (CA) experienced an increase in phylum Firmicutes, accompanied by a reduction of Bacteroides; the resulting altered microbial characteristics are analogous to the obesity-related gut microbiome. In our experiments, the F/B ratio was raised in the HFD group and down in the HQT group, and this alternating trend may be due to the excessive production of bile acids for the feeding of HFD. Besides, the genus *Blautia* was abundant in the HFD group and few in the HQT and NFD groups. *Blautia* could produce large amounts of antimicrobial secondary bile acids (BAs) from primary BAs with its 7a-dehydroxylating activity [55]. Therefore, the rich abundance of *Blautia* in the HFD group may play an important role as a compensatory response to the presence of increasing amounts of BAs in the gut [56–59]. According to the PICRUSt analysis, metabolic pathways, such as the TCA cycle, flagellar assembly, other glycan degradation, two-component system, and lipopolysaccharide biosynthesis, were enriched in the HQT group compared with the HFD group. These results were similar to the comparison between the NFD group and the HFD group. Moreover, the HQT promoted the activity of the flavonoid biosynthesis pathway. As mentioned above, the HQT contains a variety of flavonoids, such as typhaneoside, rutin, quercetin, heterosine lisu-3-o-new hesperidin, hyperoside, and isoquercetin, which must be transformed in the intestine to exert better biological activity. Thus, this result may be the key point for HQT to change the gut microbiota in NAFLD rats and can be a target for further studies.

## 5. Conclusions

In conclusion, gut dysbiosis occurs in HFD-induced NAFLD rats, and the HQT decreases lipid levels and inflammation in these NAFLD rats, together with beneficial modulation of the gut microbiota. In particular, HQT could modulate a wide range of gut microbiota, including *norank_f__Bacteroidales_S24_7_group*, *Ruminococcaceae*, *Enterobacteriaceae*, *Bifidobacterium*, *Alistipes*, *Anaeroplasma*, *Streptococcus*, *Holdemanella*, *Allobaculum*, and *Blautia*. Spearman's correlation analysis also identified that *[Ruminococcus]_gauvreauii_group*, *Lachnoclostridium*, *Blautia*, *Allobaculum*, and *Holdemanella* exhibited significant (*p* < 0.001) positive correlations with TG, CHOL, LDL-C, IL-6, IL-1*β*, TNF-*α*, and BW and negative correlations with HDL-C (*p* < 0.001). The *norank_f__Bacteroidales_S24-7_group* and *Alistipes* showed an opposite trend. Besides, according to the UHPLC-QqQ-MS analysis, the HQT contained flavonoids, such as typhaneoside, rutin, quercetin, heterosine lisu-3-o-new hesperidin, hyperoside, and isoquercetin. Interestingly, the HQT increased the KEGG pathway of flavonoid biosynthesis in fecal samples, which may be the manner in which the HQT changes the gut microbiota in NAFLD rats.

Together, these findings indicated that the effects of the HQT on NAFLD might depend on its modulatory effect on the gut microbiota.

## Data Availability

The datasets used to support this study will be made available upon request. Requests should be sent to the corresponding author.

## Supplementary Materials

Supplementary Materials Table 1: different components in the HQT formula. Table 2: the community coverage index of the NFD, HFD, and HQT groups. Figure 1: the rarefaction curves and Shannon curves of the NFD, HFD, and HQT groups. Figure 2: key bacterial alterations in response to the HFD and HQT treatments.
