# Supplementary material for: Modulation of the Gut Microbiota in Rats by Hugan Qingzhi Tablets during the Treatment of High-Fat-Diet-Induced Nonalcoholic Fatty Liver Disease
Source: Oxid Med Cell Longev. 2018 Dec 23;2018:7261619. doi: 10.1155/2018/7261619 (PMC6323444; doi:10.1155/2018/7261619)
Supplement: Supplementary Materials — Table 1: different components in the HQT formula. Table 2: the community coverage index of the NFD, HFD, and HQT groups. Figure 1: the rarefaction curves and Shannon curves of the NFD, HFD, and HQT groups. Figure 2: key bacterial alterations in response to the HFD and HQT treatments. [file 7261619.f1.pdf]

Supplementary Material :

Additional files 1: Table 1 Different components in the HQT formula.

Table 1: Herbal constituents of Hugan Qingzhi.

Pharmaceutical name	English name	Botanical name	Family	Part used	Chinese name	Ratio
Rhizoma Alismatis	Rhizome of oriental water plantain	Alisma orientalis (Sam.) Juzep.	Alismataceae	Rhizome	Ze Xie	6
Fructus Crataegi	Hawthorn fruit	Crataegus pinnatifida Bunge	Rosaceae	Fruit	Shan Zha	6
Pollen Typhae	Cattail pollen	Typha orientalis C. Presl	Typhaceae	Pollen	Pu Huang	3
Folium Nelumbinis	Lotus leaf	Nelumbo nucifera (Gaertn.)	Nymphaeaceae	Leaf	He Ye	4
Radix Notoginseng	Sanchi	Panax pseudoginseng var. notoginseng	Araliaceae	Root	San Qi	1

Additional files 2: Table 2 The community coverage index of the NFD, HFD and HQT groups.

Noted：P=prophase, M= metaphase, A=anaphase.

Sample\Estimators	coverage	Sample\Estimators	coverage	Sample\Estimators	coverage
P_NFD_79	0.995277	M_NFD_9	0.996243	A_NFD_69	0.995921
P_NFD_6	0.995707	M_NFD_69	0.994794	A_NFD_6	0.995009
P_NFD_5	0.995653	M_NFD_6	0.99415	A_NFD_5	0.995707
P_NFD_14	0.995653	M_NFD_5	0.995492	A_NFD_13	0.995492
P_NFD_13	0.995921	M_NFD_4	0.995546	A_NFD_1	0.995707
P_HQT_79	0.996458	M_HQT_79	0.996351	A_HQT_79	0.995385
P_HQT_5	0.99576	M_HQT_5	0.996404	A_HQT_7	0.996458
P_HQT_4	0.995277	M_HQT_4	0.994955	A_HQT_6	0.996458
P_HQT_14	0.996243	M_HQT_3	0.995975	A_HQT_3	0.996243
P_HQT_1	0.995331	M_HQT_14	0.996243	A_HQT_14	0.995921
P_HFD_9	0.995975	M_HFD_9	0.996082	A_HFD_79	0.995868
P_HFD_7	0.996029	M_HFD_7	0.99619	A_HFD_6	0.996458
P_HFD_6	0.995277	M_HFD_6	0.996136	A_HFD_5	0.995653
P_HFD_5	0.995975	M_HFD_5	0.996243	A_HFD_13	0.995653
P_HFD_3	0.995814	M_HFD_1	0.99678	A_HFD_0	0.997424

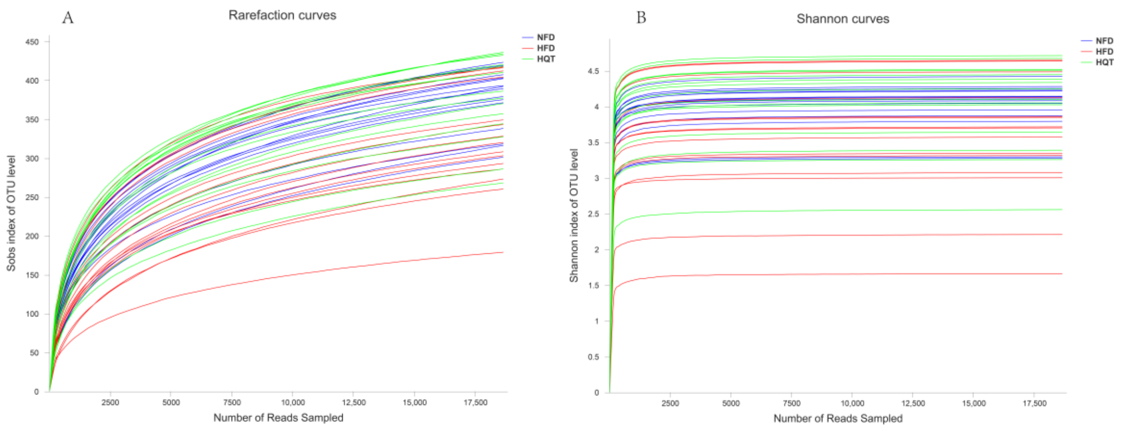

Additional files 3: Fig. 1 The rarefaction curves and Shannon curves of the NFD, HFD and HQT groups.

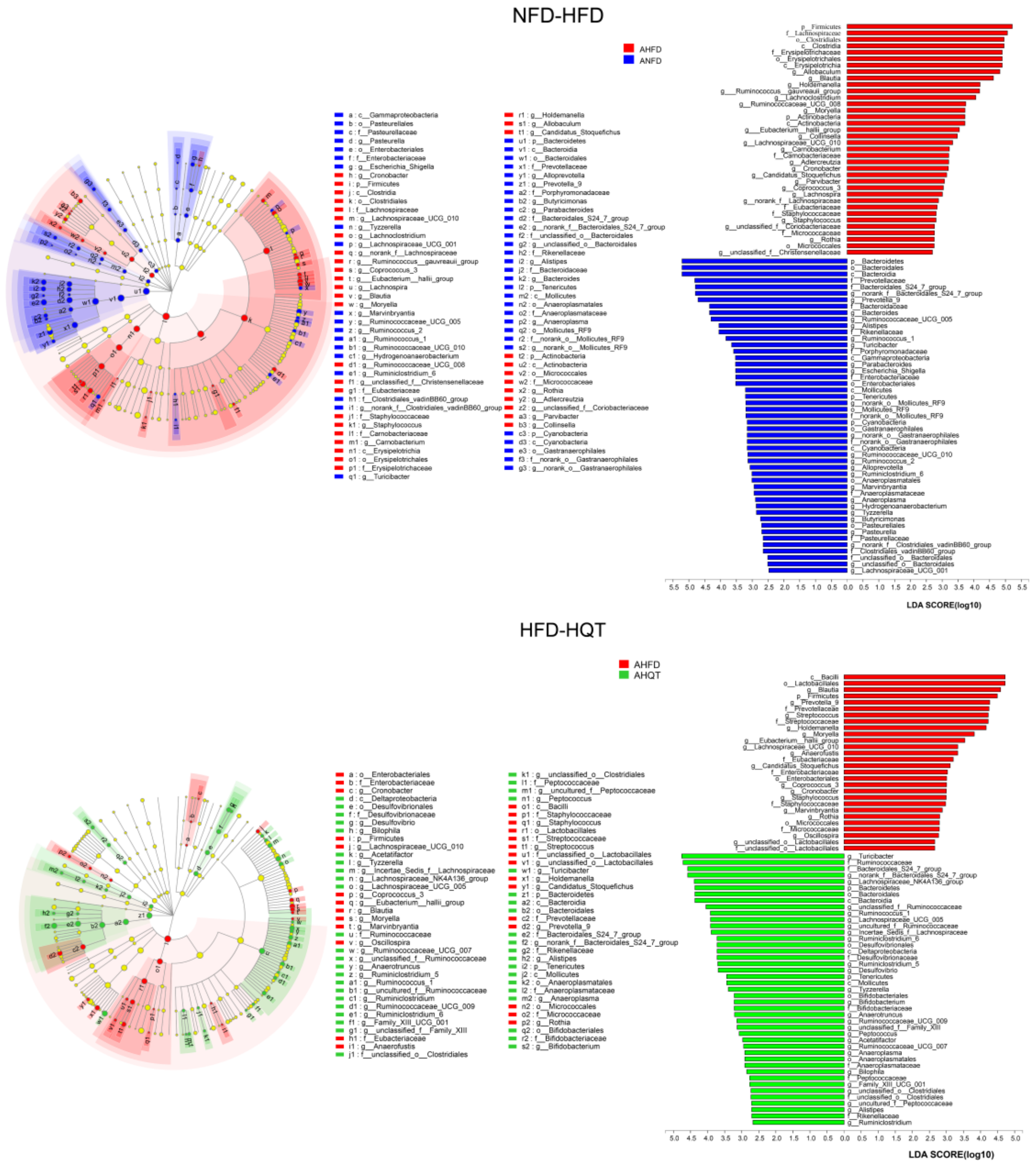

Additional files 4: Fig. 2 Key bacterial alterations in response to the HFD and HQT treatments. (A) Cladogram

generated by the LEfSe analysis (LDA=2) showing enriched taxa in 12-week feces from the NFD (blue) and HFD (red) groups. (B) LDA scores of enriched taxa shown in A. (C) Cladogram generated by the LEfSe analysis (LDA=2) showing enriched taxa in 12-week feces from the HFD (red) and HQT (green) groups. (D) LDA scores of enriched taxa shown in C.
